# Body Image and Sexual Dissatisfaction: Differences Among Heterosexual, Bisexual, and Lesbian Women

**DOI:** 10.3389/fpsyg.2019.00903

**Published:** 2019-05-09

**Authors:** Silvia Moreno-Domínguez, Tania Raposo, Paz Elipe

**Affiliations:** Department of Psychology, University of Jaén, Jaén, Spain

**Keywords:** body image, body dissatisfaction, sexual dissatisfaction, heterosexual, bisexual, lesbian, women

## Abstract

Gender-based differences in body image dissatisfaction are not conclusive. Women’s body experiences and their impact on sexual satisfaction may advance knowledge on how heterosexual, bisexual, and lesbian women internalize heterosexist values. In this study, we quantitatively examined the degree of body image and sexual dissatisfaction experienced by heterosexual, bisexual, and lesbian women, to determine whether body dissatisfaction can predict sexual dissatisfaction. Three hundred and fifty-four women completed an online survey measuring body and sexual dissatisfaction. No sexual orientation-based differences were observed in body or sexual dissatisfaction; however, body concerns were found to have less influence on sexual dissatisfaction in lesbian women compared to heterosexual and bisexual women. Standards of beauty remain constant among all women, yet removing themselves from the male gaze may be interpreted as a protective factor which shields women from expressing concern about their appearance during sexual activity.

## Introduction

There is a widespread consensus that nearly all women feel the societal pressure of having an ideal female body (Wolf, 1991; Wade and DiMaria, 2003). This pressure often translates into body image dissatisfaction, which has been labeled as normative in heterosexual women (Rodin et al., 1984). Despite the flourishing body of research, which has examined sexual orientation-based differences in women’s body image perceptions and attitudes, it remains unclear whether lesbian women experience body dissatisfaction similar to their heterosexual female counterparts. Research carried out to date points in two directions. Some studies have found that lesbian and heterosexual women share the same body issues when it comes to level of dissatisfaction (Striegel-Moore et al., 1990; Beren et al., 1996; Koff et al., 2010; Yean et al., 2013); concerns over weight and appearance (Heffernan, 1996; Yean et al., 2013); and their internalization of thin and beauty ideals (Koff et al., 2010). Given that all women in Western cultures find themselves immersed in heteronormative societies, which follow specific beauty and appearance “norms,” these findings might indicate that lesbian women are exposed to the same body dissatisfaction risks as heterosexual women (Dworkin, 1989). Other studies, meanwhile, have found that lesbian women are less influenced by oppressive beauty standards because they experience less body dissatisfaction (Siever, 1994; Conner et al., 2004; Polimeni et al., 2009; Leavy and Hastings, 2010; Alvy, 2013); hold a broader ideal of body shape (Conner et al., 2004; Alvy, 2013; Markey and Markey, 2014); are less concerned about weight and the drive for thinness (Polimeni et al., 2009; Leavy and Hastings, 2010); and possess more flexible beauty standards (Siever, 1994; Myers et al., 1999; Henrichs-Beck et al., 2015) compared to heterosexual women. In the latter case, these findings may indicate that lesbian women move within a subculture, which is likely to shield them from mainstream heteronormative beauty standards (Brown, 1987). In the classic meta-analysis conducted by Morrison et al. (2004), significant body dissatisfaction differences were observed between lesbian and heterosexual women: lesbian women were slightly more satisfied with their bodies than heterosexual women; however, the effect size was so small that it did little to clear up the issue.

The contradictory results regarding the differences between heterosexual and lesbian women in their body experiences may stem from the fact that the lesbian community is considered a homogeneous group. In a recent study, Henrichs-Beck and Szymanski (2017) suggested that lesbian gender expression within the lesbian culture may account for their body dissatisfaction differences. They found that lesbians with more stereotypically feminine traits were most at risk of body dissatisfaction, whereas lesbians with more stereotypically masculine traits, that is, “butch” women, were less likely to be affected. These findings coincide with previous literature demonstrating that greater masculinity is associated with less body dissatisfaction (Kimlicka et al., 1983; Jackson et al., 1988; Braitman and Ramanaiah, 1999), and stronger identification with femininity is related to higher body dissatisfaction (Paxton and Sculthorpe, 1991; Lakkis et al., 1999). Feminine gender expression could imply a greater risk of body dissatisfaction and drive for thinness (Meyer et al., 2001), whereas a “butch” gender expression may protect women from sexual objectification, although some research suggests that this “butch-identity” has more to do with “authenticity” and less to do with aesthetic desires (Levitt and Hiestand, 2004).

Furthermore, objectification theory has been used to explain differences between heterosexual and lesbian women in their body image concerns. Specifically, sexual objectification, which sees women more likely to be treated as sexual objects, can make them more vulnerable to developing eating disorder symptomatology. This sexual objectification is related to the internalization of the thin-ideal standard of beauty, and predisposes women to closely monitor their body image and become more body dissatisfied (Fredrickson and Roberts, 1997). Objectification theory has received robust support, primarily in relation to heterosexual women (Moradi et al., 2005; Augustus-Horvath and Tylka, 2009); however, mixed results have been found for lesbian women. Kozee and Tylka (2006) observed how higher self-surveillance levels were predictive of increased body shame, which, in turn, predicted eating disorder symptomatology in lesbian women and indicated a direct link between levels of body surveillance and negative eating attitudes. Haines et al. (2008) presented an independent replication of Kozee and Tylka’s modeled direct path between surveillance and negative eating attitudes in lesbian women, yet they also found that body self-surveillance and feeling shame about one’s appearance led to increased negative eating attitudes and depression in this group. These findings support the notion that models developed for heterosexual women may not fully apply to lesbian women. In fact, Engeln-Maddox et al. (2011) found that lesbian women share similar experiences of sexual objectification to heterosexual women, but report less body surveillance. The authors pointed to a facet of lesbian identity as something that may mitigate the effects of being objectified by others, and that the sexualized evaluation of one’s body by another woman, compared to the male gaze, could be experienced as quite different by lesbian women. Although there is limited research on sexual minority women, the findings posit that sexual objectification experienced by these minorities may differ from that of heterosexual women.

With regard to bisexuality, we know even less about bisexual women’s body experiences. Research into the influence of sexual orientation on female body image has either excluded bisexual women or grouped lesbian and bisexual women together for comparison with heterosexual women. However, because they attract both sexes, bisexual women occupy a unique social position in terms of how they move in heterosexual and LGBT cultural environments, thus deserving of individualized attention. There is increasing awareness of the distress that bisexual women may experience compared to their exclusively gay or heterosexual-identified peers. Bisexual women are interested in attracting men and may feel the pressure to fit the beauty standards defined by the male gaze. However, they are also interested in women, which can align them with lesbian women, both groups separating themselves from the dominant heterosexist culture. As a result of this dichotomy, bisexual women’s body image experiences seem to be unique. Chmielewski and Yost (2013) found that bisexual women experience a tension between their resistance to adopt sexist ideals, characteristic of feminist women, and the assumption of the thin ideal of feminine beauty. Previous studies have reported that bisexual women worry about their body image and conform to the feminine ideal (e.g., thinness) more so in relationships with men, and may enjoy greater freedom in defining their own standards of attractiveness in same-sex relationships (Huxley et al., 2011). Research also suggests that bisexual women may have more body image issues than lesbian and heterosexual women (Boehmer et al., 2007; Polimeni et al., 2009); are more prone to developing eating disorders compared to lesbian women (Koh and Ross, 2006); and exhibit higher rates of unhealthy weight-control behaviors than heterosexual women (Polimeni et al., 2009). Furthermore, research into body image and objectification theory involving sexual minority women has primarily focused on lesbian women, excluding their bisexual counterparts. To our knowledge, only one research study has analyzed the sexual objectification experiences of bisexual women. Brewster et al. (2014) examined objectification theory in bisexual women and the roles of two minority stressors, namely antibisexual discrimination and internalized biphobia. The results revealed that antibisexual discrimination and internalized biphobia yielded links with the internalization of sociocultural standards of attractiveness, body surveillance, and body shame, confirming that bisexual women’s dichotomous experiences of body image are unique and unusual.

Body image is closely related to sexual satisfaction. Feelings of body concern are shown to be associated with a negative impact on females’ daily sex lives (Wiederman and Pryor, 2000; Cash et al., 2004; Pujols et al., 2010). Research into sexual satisfaction has mostly centered on white, married, and heterosexual people; again, Lesbian, Gay, Bisexual and Transsexual groups (LGBT) have been underrepresented in these studies. Among those who identify themselves as heterosexual, earlier research confirms that body dissatisfaction is associated with increased sex-related distress and anxiety (Berman et al., 2003); feeling less comfortable exposing their bodies during sex (Cash et al., 2004); and feeling less sexual desire and engaging in less frequent sexual activity (Koch et al., 2005). As for the LGBT community, little research exists, yet findings point in the same direction. Shepler et al. (2018) found that sexual anxiety, relationship commitment, body image, and pride in identity significantly contribute to predicting sexual satisfaction in LGBT groups. Peplau et al. (2009) compared body dissatisfaction and comfort with one’s body during sexual activity among the aforementioned groups. They found that men who identify themselves as heterosexual report more positive evaluations of their appearance; less preoccupation with their weight; more positive effects of their body image on their quality of life and the quality of their sex life; and greater willingness to reveal aspects of their body to their partner during sexual activity, compared to those men who identify themselves as gay, heterosexual women, and lesbian women. These findings support the argument that gay men are at greater risk of experiencing body dissatisfaction than heterosexual men, but turn up little evidence of lesbian women’s body experiences compared to heterosexual women. Measures of body satisfaction in lesbian and heterosexual women did not differ significantly, with similarities observed in mean *self-rated attractiveness* and *comfort in a swimsuit*, although there was a small trend for more heterosexual women than lesbian women to report hiding at least one aspect of their bodies during sex (52% vs. 44%), especially their stomach. Regarding bisexuality, a more positive body image was found to be associated with higher levels of sexual activity and sexual satisfaction in bisexual women (Zamboni et al., 2007).

Women’s body experiences and the consequences they have on their sexual satisfaction give us a good opportunity to analyze the impact of body image concerns on female quality of life. Findings from previous literature suggest that the more dissatisfied a woman is with her own body, the more likely she is to report a dissatisfying sex life. However, the scale of this effect may be influenced by sexual orientation. It is during sexual activity when the body is completely exposed to the “others gaze.” As such, making comparisons about how body image concerns impact on sexual satisfaction depends on whether the other gaze comes from a woman or a man. The outcomes may yield knowledge about the pressures to fit the beauty standards defined by the male gaze and about the internalization of heterosexist values among heterosexual, lesbian, and bisexual women. Past literature studying the role of body image on sexual satisfaction has focused primarily on heterosexual samples, and the few research studies published on sexual minority women have either compared lesbian and heterosexual women—excluding bisexual women—or have solely focused on bisexual women. To our knowledge, no studies to date have analyzed this topic by simultaneously comparing heterosexual, lesbian, and bisexual women. Thus, our study aimed to quantitatively examine the degree of body image and sexual dissatisfaction experienced by heterosexual, lesbian, and bisexual women to determine (1) whether body dissatisfaction is a reliable predictor of sexual satisfaction; and (2) whether differences by sexual orientation exist. In line with earlier research, we hypothesized that bisexual women would report less dissatisfaction with their bodies compared to their heterosexual and lesbian counterparts, and lesbian women would be slightly more satisfied with their bodies than heterosexual women. In previous studies, lesbian, bisexual, and heterosexual women reported more similarities than differences in their sex life (Matthews et al., 2002; Henderson et al., 2009). Therefore, we did not expect to find differences in sexual satisfaction and frequency of sexual activity by sexual orientation. Finally, objectification theory has been widely confirmed in all women, including sexual minority groups, although lesbian and bisexual women have yielded mixed and more complex results. Therefore, we hypothesized that women interested in attracting men (heterosexual and bisexual) would feel more pressure to fit the beauty standards defined by the male gaze, and that their body concerns would have the most impact on their sexual satisfaction, compared to women removed from the male gaze in their sexual relations (lesbian women).

## Materials and Methods

### Participants

Three hundred and fifty-four (354) women were initially recruited into the study. However, the final sample was reduced to 333 participants after removing 21 women (13 were underage and eight left some responses blank). *A priori* analyses of power were run to select a sufficient number of participants according to our chosen statistical test. Based on the results for detecting a small-to-medium effect size (0.18), with a statistical power of 0.8, a minimum of 303 participants would be required. The women were asked to complete a survey prompted by online advertisements on different websites. Postings were added to general community forums and websites of interest to lesbian and bisexual women in Spain. Participation was voluntary and completely anonymous; no compensation was offered to survey respondents. A total of 176 women identified themselves as heterosexual; 79 as bisexual; and 78 as lesbian. Ages ranged from 18 to 62 years, with a mean age of 25.43 (*SD* = 7.21). The participants’ educational level ranged from “no high-school qualifications” to “higher education” (university-level studies).

### Measures

#### Demographic Variables

Participants reported age, educational level, height (in centimeters), weight (in kilograms), relationship status, sexual orientation, and frequency of sexual activity via checkboxes. Relationship status responses were elicited through the item “Currently…” with three options to choose from: “I am in a stable relationship”; “I am casually dating”; and “I am not dating anyone.” Respondents were asked about their sexual orientation in the following way: “I consider myself…” and the options: “Heterosexual”; “Homosexual”; “Bisexual.” Taking into account the past 6 months, frequency of sexual activity was elicited via the statement: “I have sex...” and four possible responses: “Daily”; “A few times a week”; “A few times a month; and “I haven’t had sex.”

Body Mass Index (BMI) was calculated as a continuous variable using the standard formula of kilograms over height squared.

The Body Shape Questionnaire (BSQ; Cooper et al., 1987) was used to assess body dissatisfaction. This 34-item self-report instrument measures the degree of concern with body image, especially feelings of fatness (e.g., “Have you thought that your thighs, hips or bottom are too large for the rest of you?”). Respondents rate each item on a scale of 1–6 (1 = *Never*, 6 = *Always*). The total score ranges from 34 to 204, and scores above 105 are indicative of mild to severe body dissatisfaction. The BSQ has been validated with Spanish-speaking populations (Raich et al., 1996). This questionnaire shows a high consistency for measuring body dissatisfaction (Vázquez et al., 2011). In the present study, Cronbach’s alpha was 0.97.

The Index of Sexual Satisfaction (ISS; Hudson, 1998) was used to assess sexual dissatisfaction. This 25-item questionnaire measures current partners’ sexual dissatisfaction with their sexual interactions (e.g., “I feel that my sex life is boring”). Respondents rate each item on a scale of 1–5 (1 = *Never*, 5 = *Always*). Some items are worded positively and coded inversely. All 25 items are summed after the positively worded items have been reverse scored and then subtracting 25 from this sum, giving the total score. The scores range from 0 to 100, and those equal to or greater than 30 indicate sexual dissatisfaction. The ISS has been adapted into Spanish (Crooks and Baur, 2000) and validated with Spanish-speaking populations, showing a high consistency for measuring sexual dissatisfaction (Iglesias et al., 2009). In the present study, Cronbach’s alpha was 0.90.

### Procedure

We used the same procedure as described in Henrichs-Beck and Szymanski (2017). Participants were recruited through postings placed on several Facebook pages. The ads featured a brief explanation of the study and a link to the online survey. These ads were shown to Facebook users who indicated they were over 18, lived in Spain and have Spanish nationality, and identified themselves as female. Postings appeared on pages of general interest and pages of specific lesbian interest (e.g., LGTBI associations or private groups in Facebook) Some participants were also recruited by asking them to directly forward the research announcement. Hence, a snowballing sampling technique was employed, whereby links were posted on social media networking sites or emailed to contacts, and respondents were asked to share them with their networks. We used the survey platform Eval&Go. Our study was carried out in accordance with the recommendations outlined in the APA Ethical Guidelines for Human Research and adhering to the principles of Spain’s Organic Law 15/1999 for the Protection of Personal Data (Legislación Consolidada, 1999). Before starting the survey, participants were warned that it contained information of a sexual nature. They were also informed that participation was voluntary and anonymous, and a guarantee was given that the data would only be used for research purposes. Participants consented to completing the survey and confirmed that they understood the purpose of the study. The survey comprised 69 items corresponding to the chosen questionnaires. Participants were of adult age (>18 years); they received the necessary information to help them decide whether to participate or not; and there were no potential risks associated with the research, therefore the approval of the ethics committee was not applicable according to the institutional ethical committee and national guidelines. All participants signed informed consent in accordance with the Declaration of Helsinki.

### Data Analyses

A contingency table and a chi-squared test were used to analyze the correlation between frequency of sexual activity, relationship status, and sexual orientation. One-way ANOVAs were performed to determine differences in body dissatisfaction, sexual dissatisfaction, and BMI among heterosexual, bisexual, and lesbian women. In order to examine how the variables interrelate and whether they relate differently depending on sexual orientation, we tested for bivariate correlations between them for each sexual orientation group using Pearson’s correlation coefficient for the BSQ score and BMI, and Spearman’s rho for frequency of sexual activity because of its ordinal nature. Furthermore, we ran a Fisher’s r-to-z transformation to compare correlation indices for each correlation pair (heterosexual vs. lesbian; heterosexual vs. bisexual; lesbian vs. bisexual). Finally, simultaneous multiple regression analyses were performed for each sexual orientation to confirm the predictive value of all independent variables regarding sexual dissatisfaction. This regression method is recommended when no specific predictions are made about the weight of each variable, as in this case. However, there is interest in determining the degree of influence of several variables and the relative influence of each variable under study (Keith, 2006, p.76). The analyses were carried out using statistical package IBM SPSS version 24.0. We used the G^∗^Power software (Faul et al., 2007) to calculate statistical power.

## Results

Regarding frequency of sexual activity, the contingency table (see Table 1) reveals similar percentages of sexual activity in women by sexual orientation. The chi-squared analyses confirmed no significant correlations between variables[χ^2^_(6,333)_ = 6.19, *p* = 0.40].

**Table 1:** Frequency of sexual activity by sexual orientation.

	Never (*n* = 23)	Few times/month (*n* = 94)	Few times/week (*n* = 188)	Daily (*n* = 28)
Heterosexual	5.1%	29.0%	57.4%	8.5%
Homosexual	5.1%	26.6%	58.2%	10.1%
Bisexual	12.8%	28.2%	52.6%	6.4%

In terms of relationship status, 75.4% of women reported having a stable partner; 13.8% said they were casually dating; and 10.8% were not dating anyone. Again, no significant correlation between sexual orientation and relationship status was found [χ^2^_(4,333)_ = 8.02, *p* = 0.91].

Given that the primary aim was to determine whether body dissatisfaction might predict sexual dissatisfaction, women who reported not having had sex in the last 6 months were excluded from all remaining analyses. Therefore, the following analyses were run on 310 women.

The one-way ANOVA results showed no significant differences among heterosexual, bisexual, and lesbian women in terms of body dissatisfaction (BSQ), sexual dissatisfaction (ISS), and BMI (see Table 2). Age^1^ was also analyzed to check for possible between-group differences, yet no significant differences were found.

**Table 2:** Descriptive statistics of BSQ, ISS, BMI scores and age, by sexual orientation.

		*M*	*SD*	Min	Max	*F*	*p*
BSQ^1^	Heterosexual	88.87	36.49	36.00	182.00		
	Lesbian	88.76	41.04	34.00	183.00	0.52	0.56
	Bisexual	88.38	39.08	34.00	204.00		
ISS^2^	Heterosexual	18.96	11.64	2.00	53.00		
	Lesbian	18.56	11.83	0.00	58.00	0.62	0.94
	Bisexual	18.44	11.55	0.00	57.00		
BMI^3^	Heterosexual	24.40	5.15	16.00	42.54		
	Lesbian	25.78	6.51	16.90	51.60	1.80	0.17
	Bisexual	24.40	4.91	16.96	38.86		
AGE	Heterosexual	24.91	0.57	18.00	57.00		
	Lesbian	26.47	0.74	18.00	57.00	1.26	0.28
	Bisexual	25.72	0.93	18.00	62.00		

*^1^Body Shape Questionnaire. ^2^Index of Sexual Satisfaction. ^3^Body Mass Index.*

The correlation coefficients of the associations between variables which were later entered into the regression analysis for each group were calculated (See Table 3).

**Table 3:** Correlations between BSQ and BMI scores (Pearson) and frequency of sexual activity (Spearman), with sexual dissatisfaction (ISS) by sexual orientation.

	Heterosexual (*n* = 167)	Lesbian (*n* = 75)	Bisexual (*n* = 68)
BSQ	0.35^∗∗a^	0.17	0.12^a^
BMI	0.25^∗∗a^	0.20^b^	−0.15^a,b^
Frequency of sexual activity	−0.42^∗∗^	−0.33^∗∗^	−0.27^∗^

*^∗^p < 0.05; ^∗∗^p < 0.01. ^a,b^Matched superscript across rows are those between which are significant differences according to Fisher’s r-to-z transformation. Numbers without superscript point to an absence of significant differences with the rest.*

The results revealed that heterosexual women were the only group in which sexual dissatisfaction was significantly correlated with BSQ scores, BMI, and frequency of sexual activity, indicating that increased body dissatisfaction, high BMI, and low frequency of sexual activity are associated with less sexual satisfaction. In contrast, in bisexual and lesbian women, only frequency of sexual activity was significantly correlated with sexual dissatisfaction, demonstrating how less sexual activity on its own is associated with less sexual satisfaction. Fisher’s r-to-z transformation showed significant differences in the BSQ–sexual dissatisfaction correlation between heterosexual and bisexual women (*z* = 1.67, *p* < 0.05), yielding a stronger correlation in heterosexual women as well as in the BMI–sexual dissatisfaction correlation between heterosexual and bisexual women (*z* = 2.77, *p* < 0.005) and between lesbian and bisexual women (*z* = 1.89, *p* < 0.05), stronger in the first instance.

Simultaneous multiple regression analyses were conducted to predict sexual dissatisfaction by taking into account body dissatisfaction, BMI and frequency of sexual activity as predictors. Given the ordinal nature of frequency, three dummy variables were created: daily, weekly, and monthly.

Different result patterns emerged for each sexual orientation. For heterosexual women, a model explaining 27.0% of the variance was obtained, *F*(4,164) = 16.18, *p* < 0.001. Body dissatisfaction and monthly sexual activity were significant in terms of predicting sexual dissatisfaction, with the most relevant predictor being monthly sexual activity. In lesbian women, a significant model accounting for 20.2% of the variance was obtained, *F*(4,73) = 5.63, *p* = 0.001; however, in this case, only monthly sexual activity significantly predicted sexual dissatisfaction. The analysis of bisexual women generated a model explaining 13.4% of the variance, *F*(4,67) = 3.60, *p* = 0.011. For bisexual women, body dissatisfaction, BMI, and monthly sexual activity were the variables that significantly predicted sexual dissatisfaction. See Table 4 for the parameter estimates in the analyses.

**Table 4:** Parameter estimates in the multiple regression analyses for heterosexual, lesbian, and bisexual women.

		B	S.E.	β	*t*	Sig.	C.I. 95%	
Heterosexual (*n* = 167)	Constant	2.70	3.83		0.71	0.48	−4.86	10.28
	BSQ	0.08	0.03	0.24	3.14	0.00	0.03	0.13
	BMI	0.28	0.17	0.12	1.60	0.11	−0.07	0.62
	Monthly frequency	9.50	1.72	0.38	5.51	0.00	6.09	12.90
	Daily frequency	−3.00	2.87	−0.07	−1.05	0.30	−8.68	2.67
Lesbian (*n* = 75)	Constant	5.05	5.24		0.96	0.40	−5.406	15.51
	BSQ	0.04	0.03	0.15	1.27	0.21	−0.024	0.10
	BMI	0.28	0.21	0.15	1.31	0.20	−0.147	0.71
	Monthly frequency	10.69	2.83	0.41	3.78	0.00	5.044	16.33
	Daily frequency	−4.25	4.24	−0.11	−1.00	0.32	−12.721	4.21
Bisexual (*n* = 68)	Constant	24.30	6.74		3.60	0.00	10.82	37.77
	BSQ	0.09	0.04	0.31	2.41	0.02	0.02	0.17
	BMI	−0.68	0.30	−0.29	−2.27	0.03	−1.28	−0.08
	Monthly frequency	8.70	2.90	0.36	3.00	0.00	2.91	14.50
	Daily frequency	1.91	5.12	0.04	0.37	0.71	−8.32	12.13

*Criterion variable: Sexual dissatisfaction (Index of Sexual Satisfaction). Weekly frequency was excluded from all regression analyses. BSQ: Body Shape Questionnaire; BMI: Body Mass Index.*

## Discussion

To our knowledge, the present study is the first to quantitatively examine differences in body image and sexual dissatisfaction by sexual orientation, and to determine whether body concerns might predict sexual satisfaction in heterosexual, lesbian, and bisexual women.

Findings revealed no differences in levels of body dissatisfaction among lesbian, bisexual, and heterosexual women in line with our predictions. However, based on the premise that sexual orientation may be a protective factor against the negative influence of male gaze-based beauty standards, the hypothesis that heterosexual and bisexual women’s body concerns would have the greatest impact on their sexual satisfaction, compared to lesbian women, was confirmed. As expected, no differences in sexual dissatisfaction and frequency of sexual activity by sexual orientation were found.

A quantitative examination of the body dissatisfaction differences revealed no sexual orientation-based significant differences. Contrary to our expectations, overall levels of body dissatisfaction were similar in all three groups of women This result coincides with those studies in which no overall differences in level of satisfaction between lesbian and heterosexual women were observed (Striegel-Moore et al., 1990; Beren et al., 1996; Heffernan, 1996; Morrison et al., 2004; Grogan et al., 2006; Peplau et al., 2009; Koff et al., 2010; Yean et al., 2013; Huxley et al., 2014; Tiggemann, 2015), yet it also contradicts research suggesting that bisexual women may experience more body image issues than their lesbian and heterosexual peers (Koh and Ross, 2006; Boehmer et al., 2007; Polimeni et al., 2009). The absence of significant differences between lesbian, bisexual, and heterosexual women in body dissatisfaction levels could indicate that the pressure to fit beauty standards is widespread across all women, regardless of their sexual orientation (Wolf, 1991). Nearly all women are considered successful in various life domains based on their appearance and thinness ideals (Heilman and Stopeck, 1985; Wade and DiMaria, 2003). Body evaluation is central to sexual objectification and a woman’s appearance is constantly being scrutinized by men and other women. This, therefore, may explain the normative levels of body dissatisfaction reported by the female population, including sexual minority women. The media promotes a thin and curvaceous ideal female body shape, and bisexual and lesbian women experience similar mainstream pressures to conform to this thin ideal; therefore, the similarities regarding the overall level of body dissatisfaction among heterosexual, lesbian, and bisexual women reported in the present study are justified (Smith et al., 2017).

Our study also confirms that the internalization of heterosexist values defined by the male gaze may prove relevant to identifying the scale of pressure experienced by women of different sexual orientations. After examining the links between body concerns and sex dissatisfaction, we found that both constructs were significant and differently correlated across all groups of women. Correlation analyses revealed that less frequent sexual activity is associated with less sexual satisfaction in all women, while higher levels of body dissatisfaction and BMI and less frequent sexual activity are associated with less sexual satisfaction in heterosexual women only. Furthermore, the multiple regression analyses showed that body dissatisfaction predicts sexual dissatisfaction in heterosexual and bisexual women, but not in lesbian women. In heterosexual women, the regression model was the most explanatory (accounting for almost 30% of the variance of sexual dissatisfaction); frequency of sexual activity and body dissatisfaction were significant, but not BMI. In bisexual women, the regression model explained the least variance compared to the other two groups, but all independent variables predicted sexual dissatisfaction, whereas in lesbian women, only frequency of sexual activity predicted sexual dissatisfaction. Starting from the premise of objectification theory, we hypothesized that the sexual satisfaction of women interested in attracting men (heterosexual and bisexual women) would be more affected by their body concerns compared to those removed from the male gaze in their sexual relations (lesbian women). These findings, taken together, indirectly support this premise. In general terms, they coincide with the study published by Peplau et al. (2009), in which lesbian and heterosexual women did not differ significantly in body dissatisfaction, although there was a small trend for heterosexual women to hide some parts of their body during sex.

It could be argued that women sensitive to the male gaze may have strongly internalized heterosexist values about beauty standards and, as a consequence, their body concerns may have more strongly affected their sex lives. Therefore, sexual orientation may be a protective factor for women who are not likely to be judged by the man-defined “attractiveness” standard of beauty. Somehow lesbian women still seem to retain this kind of protective factor with regard to their body concerns, at least when it comes to their sexual activity. Appearance is important in sexual relationships for both women and men (Cash et al., 2004). Exclusive lesbians do not engage in sexual relations with men, and this holds partially true for bisexual women. Because lesbians seem to be freer from the male gaze, it could be argued that societal pressure to be physically attractive is less salient for lesbian women, as Brown (1987) pointed out. Some authors confirm that men value physical attractiveness more than women do. In a large-scale survey, heterosexual and gay men considered appearance and face attractiveness quite important, whereas women ranked personality traits as more relevant than good looks (Lippa, 2007). The women in Markey and Markey’s (2006) study believe that men have a strong preference for attractiveness and thinness, and expect them to meet these standards. Legenbauer et al. (2009) found that men, in particular, prefer an attractive partner regardless of their sexual orientation. Interestingly, no differences between lesbian and heterosexual women were observed in levels of body dissatisfaction or internalization of beauty standards; however, a lesbian’s preference for attractiveness and thinness in a partner was not influenced by their body shape or weight dissatisfaction. The authors concluded that lesbian women who felt body dissatisfaction were the least affected; they did not expect their respective partners to be excessively critical about their appearance as a male partner would be toward heterosexual women. Smith et al. (2017) also found that lesbian women were not necessarily protected from mainstream body ideals and appearance norms, despite of their perceptions about being more accepting of larger body types and alternative styles by their partners. In line with these results, our study findings suggest that lesbian women are not shielded from experiencing body dissatisfaction, but they are in terms of how body concerns affect other life domains, such as sexual experiences.

Neither heterosexual nor bisexual women enjoy this kind of protection. Our results indicate increased distress among bisexual women compared to exclusively gay or heterosexual women, as the literature shows (Koh and Ross, 2006; Boehmer et al., 2007; Polimeni et al., 2009). Bisexual women represent the only group in which the three main independent variables (body dissatisfaction, BMI, and frequency of sexual activity) predict sexual dissatisfaction. Therefore, it could be argued that sexual satisfaction in bisexual women may be more negatively affected by their body concerns. However, an unexpected result emerged for BMI: higher levels of body dissatisfaction and low sexual activity frequency predict sexual satisfaction, whereas a lower BMI predicts increased sexual dissatisfaction. Data about the role of BMI in the body dissatisfaction– sexual orientation relationship are equivocal. Some studies have found that BMI is more strongly associated with body dissatisfaction in heterosexual women than in lesbian or bisexual women (e.g., Share and Mintz, 2002), whereas others have not drawn this conclusion (e.g., Peplau et al., 2009). Nevertheless, Davids and Green (2011), in a study focused on bisexual population, found that BMI and self-esteem were predictors of body dissatisfaction in bisexual women versus lesbian and heterosexual women. These could mean that bisexual women could be more vulnerable to the influence of BMI in body dissatisfaction, which could affect to sexual dissatisfaction. On the other hand, although the direction of the association ‘the higher the BMI, the more body dissatisfaction’ is unequivocal in the literature, some studies have found non-linear relationship between BMI and psychopathology. For instance, the results of the study by De Wit et al. (2009) give evidence for a significant U-shaped trend in the association between BMI and depression. Same results were found regarding association between BMI and anxiety (DeJesus et al., 2016). In a similar way, some studies have reported inverse or inverted J-shaped relationship between BMI and completed suicide, such that suicide risk is highest among individuals with BMI < 20 kg/m^2^ (and, ≥35 kg/m^2^, in those that report a J-shaped relationship), with lowest risk in the overweight and moderate obesity range (Dutton et al., 2013; Gao et al., 2013; Brown et al., 2018). Thus, and being cautious because we have not assessed psychopathology, maybe a non-linear relationship which the regression model does not reflect, could explain these results. It is clear that more research is necessary to clarify the role that BMI plays in bisexual women’s body dissatisfaction. In line with Swami and Tovée (2006), we found no BMI differences among the three groups of women under study, unlike some studies that have observed a higher BMI in lesbian women than in heterosexual women (Brand et al., 1992; Herzog et al., 1992; Koff et al., 2010). However, if heterosexual, bisexual, and lesbian women experience similar mainstream pressures to conform to the thin and curvaceous body ideal, then we would expect to observe similarities in their BMI. Special attention would also need to be given to determining whether BMIs tend to equal out among sexual minorities in future studies.

Certain limitations have been identified in our study. We had to shorten the survey in order to increase participation; consequently, no additional measures beyond body and sexual dissatisfaction were included. Eating or sexual disorders were not taken into account, nor were measures of relationship satisfaction, which have been found to mediate the link between body satisfaction and sexual satisfaction in lesbian, bisexual, and heterosexual women (Markey and Markey, 2014). Furthermore, considering additional protective and risk factors regarding body dissatisfaction—including, for example, feminine and masculine gender expressions and the internalization of beauty and thinness ideals—would be welcome in futures studies. Because participants were recruited over the Internet, the sample was not fully representative. The socio-economic status of participants was not assessed in the present study: given that the survey was online, participants were women that have access to technology and time to complete the survey, which may compromise the representativeness of the present sample. A certain recruitment bias could be that non-Internet users were not included in the sample. Another bias relates to the sites where the survey was posted: by resorting to specific lesbian-interest web pages, some women from the lesbian community may have been overrepresented, especially those holding a more assertive profile regarding their sexual orientation. Finally, other minorities such as transgender or non-binary persons, were not included in the present study. Determining whether the conclusions we have drawn are applicable to the aforementioned groups could be a valuable next step in future research endeavors.

Our study also has several strengths. It represents one of few studies examining differences in body dissatisfaction experience by sexual orientation, encompassing sexual minorities such as lesbians and bisexuals. The differences observed between bisexual and lesbian women indicate that all orientations should be considered, and confirms the importance of including bisexuality in future studies. The absence of differences among lesbian, bisexual, and heterosexual women in the quantitative levels of body dissatisfaction in this and previous studies could be indicative of how factors traditionally seen to protect lesbian women are compromised. Lesbian women are less likely to be judged by male-defined standards of beauty; however, the slight distinction between lesbian and straight female looks could cause traditional beauty standards to become widespread in the lesbian community. A trend toward the homogenization of the beauty canon could trigger an increase in body dissatisfaction levels in women, regardless of their sexual orientation. This study highlights the need to regulate the abuse of the dominant aesthetic canon that harms women of all sexual orientations and identities.

To conclude, our study aimed to quantitatively examine the degree of body image and sexual dissatisfaction experienced by heterosexual, lesbian and bisexual women, to determine whether body dissatisfaction can predict sexual dissatisfaction. No sexual orientation-based differences were found for body dissatisfaction, frequency of sexual activity, relationship status, or sexual dissatisfaction. However, body dissatisfaction did exert a lesser influence on sexual dissatisfaction in lesbian women compared to their heterosexual and bisexual counterparts. The fact that lesbian women are uninterested themselves in the male gaze could be interpreted as a protective factor which shields lesbian women from the consequences of feeling concerned about their appearance. The traditional beauty standard may become widespread among all women, confirming the need to regulate the abuse of the dominant aesthetic canon.

## Ethics Statement

This study was carried out in accordance with the recommendations of the Ethical Commitment of the University of Jaén with written informed consent from all subjects. All subjects gave written informed consent in accordance with the Declaration of Helsinki. The protocol was approved by the University of Jaén.

## Author Contributions

SM-D, TR, and PE designed the study. SM-D and TR collected the data. SM-D and PE conducted the analyses in close consultation with TR. SM-D, TR, and PE wrote the first draft of the manuscript. All authors contributed to and have approved the final manuscript.

## Conflict of Interest Statement

The authors declare that the research was conducted in the absence of any commercial or financial relationships that could be construed as a potential conflict of interest.

## Footnotes

1 Given that age was not a primary outcome, it was not included in the regression analyses.
